# Determinants of cardiac output in health and heart failure

**DOI:** 10.1113/EP091505

**Published:** 2025-03-23

**Authors:** Ibrahim Ainab, Natalie Van Ochten, Emmett Suckow, Kathryn Pierce, Chelsea Arent, Joseph Kay, Lindsey M. Forbes, William K. Cornwell

**Affiliations:** ^1^ Department of Medicine‐Cardiology University of Colorado Anschutz Medical Campus Aurora Colorado USA; ^2^ Department of Medicine University of Colorado Anschutz Medical Campus Aurora Colorado USA; ^3^ Clinical Translational Research Center University of Colorado Anschutz Medical Campus Aurora Colorado USA; ^4^ Department of Medicine, Division of Pulmonary and Critical Care Medicine University of Colorado Anschutz Medical Campus Aurora Colorado USA

**Keywords:** cardiac output, exercise physiology, heart failure, oxygen uptake

## Abstract

Sustained physical exercise depends on delivery of oxygenated blood to exercising muscle. At least among healthy individuals, bulk transport of blood is tightly matched to metabolic demand, such that cardiac output increases by ∼6 L/min for every 1 L/min increase in oxygen uptake. Multiple factors contribute to the regulation of cardiac output, including central command, the exercise pressor reflex (EPR) and arterial baroreceptors. Pulmonary arterial and left ventricular pressures increase in proportion to the rise in cardiac output and exercise intensity. The right ventricle augments contractility to maintain ventricular–arterial (VA) coupling and lusitropy to facilitate venous return. Among patients with heart failure (HF), however, the ability to deliver blood to exercising muscle is compromised as a result of multiple abnormalities impacting EPR, ventricular contractility, haemodynamics and VA coupling. The purpose of this review is to provide an overview of the factors limiting exercise capacity and cardiac output among patients with HF compared to what is known about normal physiology among healthy individuals.

## Abstract (highlights)

- **What is the topic of this review?**
  This review article highlights the determinants of cardiac output among healthy individuals and those with heart failure (HF), as well as the relationship between cardiac output and oxygen uptake (${\dot{V}}_{O_{2}}$). The oxygen cascade is reviewed, along with abnormalities that occur in this multi‐step process that contribute to limitations in functional capacity among patients with HF.
- **What advances does it highlight?**
  This review emphasizes novel insights into cardiovascular haemodynamics and cardiac output in healthy individuals and those with HF, derived from contemporary invasive haemodynamic analyses.

## 1 INTRODUCTION

The ability to perform and sustain physical work requires transport and utilization of oxygen to exercising muscle. This mobilization of atmospheric oxygen, from the environment to the mitochondria, is described by the ‘oxygen cascade’ (Rudofker et al., 2025; Sarma & Levine, 2018; Treacher & Leach. R.M., 1998). The Fick principle, first described by Adolf Eugen Fick in 1870, describes the relationship between oxygen uptake (${\dot{V}}_{O_{2}}$) during exercise and its overall determinants, including a central component (i.e., cardiac output; ${\dot{Q}}_{C}$), a peripheral component (i.e., oxygen uptake in the periphery; $A{\dot{V}}_{O_{2}\mathrm{diff}}$) and the oxygen shuttle (i.e., haemoglobin; Hgb). Following the diffusion of oxygen across the pulmonary capillaries, the heart and cardiovascular system are responsible for the bulk transport of oxygen to the skeletal musculature. The delivery of oxygen is regulated by multiple factors, including cerebrovascular and autonomic reflexes, intrinsic performance of the right and left (LV) ventricles, as well as peripheral dilatation of exercising vascular beds and vasoconstriction of non‐exercising beds. A breakdown in the design of this system, such as occurs with heart failure (HF), leads to impairments in health‐related quality‐of‐life (HRqOL) and functional capacity, as well as an increase in HF hospitalizations and overall mortality. Among patients suffering from HF, a breakdown in the oxygen cascade may occur at multiple levels, disrupting the relationship between ${\dot{Q}}_{C}$ and ${\dot{V}}_{O_{2}}$. The purpose of this review is to discuss normal physiological responses to exercise with an emphasis on cardiovascular performance during submaximal and peak exercise, as well as cardiovascular pathophysiology in the setting of HF.

## 2 THE FICK PRINCIPLE, OXYGEN UPTAKE AND THE OXYGEN CASCADE

**FIGURE 1:**
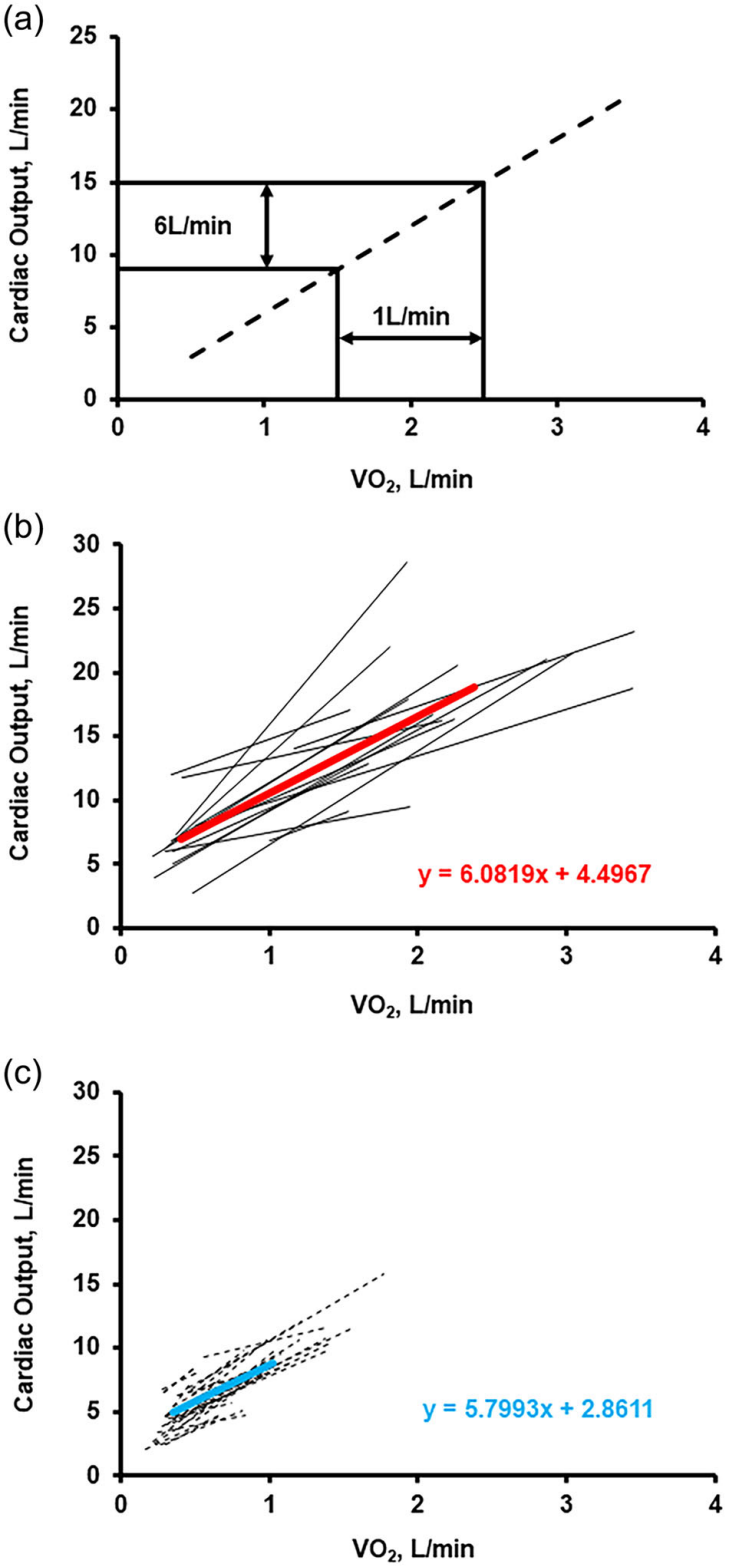
(a) The relationship between cardiac output (${\dot{Q}}_{C}$) and oxygen uptake (${\dot{V}}_{O_{2}}$) during exercise. Normally, ${\dot{Q}}_{C}$ increases by 5–6 L/min for every 1 L/min increase in ${\dot{V}}_{O_{2}}$. (b) ${\dot{Q}}_{C}$:${\dot{V}}_{O_{2}}$ slope among healthy individuals (*n* = 15) undergoing invasive cardiopulmonary exercise testing (CPET). Continuous black lines represent individual data. Continuous red‐line indicates group‐averaged data with mean slope of 6.0819; (c) ${\dot{Q}}_{C}$:${\dot{V}}_{O_{2}}$ slope among patients with HFrEF (*n* = 36) undergoing invasive CPET. Dashed black lines represent individual data. Continuous blue‐line indicates group‐averaged data with mean slope of 5.7993. See text for further details. Data provided from laboratory of senior author (W.K.C.). HFrEF, HF with reduced ejection fraction.

From a qualitative standpoint, the Fick equation describes the factors contributing to ${\dot{V}}_{O_{2}}$, namely ${\dot{Q}}_{C}$ and $A{\dot{V}}_{O_{2}\mathrm{diff}}$. According to Fick, ${\dot{V}}_{O_{2}}$ is determined by the following equation:

$${\dot{V}}_{O_{2}}={\dot{Q}}_{C}\times \mathrm{Hgb}\times \;A{\dot{V}}_{O_{2}\mathrm{diff}},$$

where ${\dot{V}}_{O_{2}}$ is oxygen uptake, ${\dot{Q}}_{C}$ is cardiac output, Hgb is haemoglobin and $A{\dot{V}}_{O_{2}\mathrm{diff}}$ is oxygen uptake in the periphery. This equation demonstrates that the ability to increase ${\dot{V}}_{O_{2}}$ and engage in and sustain physical work requires delivery of oxygenated blood by the cardiovascular system, as well as uptake and utilization of oxygen at the level of exercising muscle in the periphery. Said another way, ${\dot{V}}_{O_{2}}$ is determined by a central component (${\dot{Q}}_{C}$) and a peripheral component ($A{\dot{V}}_{O_{2}\mathrm{diff}}$). The relationship between ${\dot{Q}}_{C}$ and ${\dot{V}}_{O_{2}}$ is independent of age and sex and remains firm across the spectrum of cardiovascular diseases, such that ${\dot{Q}}_{C}$ increases by 5–6 L/min for every 1 L/min increase in ${\dot{V}}_{O_{2}}$ (Figure 1) (Proctor et al., 1998; Rowell, 1986). Among patients with HF, this relationship may or may not be preserved due to central factors such as impaired contractile reserve, and/or peripheral factors including impaired oxygen transport and utilization (Edward, Banchs, et al., 2023, Edward, Parker, et al., 2023; Skow et al., 2024). At the extremes of HF, for example, among patients with impending decompensation, ${\dot{Q}}_{C}$ may only increase by 3–4 L/min for every 1 L/min increase in ${\dot{V}}_{O_{2}}$, signifying a dramatic impairment in the ability of the heart to pump blood to the body.

The oxygen cascade defines the step‐by‐step pathway by which atmospheric oxygen is mobilized to exercising muscle in order to facilitate generation of ATP in the mitochondria and sustain exercise. This cascade is defined by the following factors or steps (Huang et al., 2022; Rudofker et al., 2025; Sarma & Levine, 2018; Treacher & Leach. R.M., 1998):

1. Atmospheric partial pressure of oxygen.
2. Interface between the environment and body at the level of the lung and ventilation.
3. Diffusion of oxygen from alveoli into pulmonary capillaries and blood.
4. Binding of oxygen to Hgb.
5. Delivery of oxygen to the exercising muscle by the cardiovascular system.
6. Diffusion of oxygen into the skeletal muscle through skeletal muscle capillaries.
7. Oxidative phosphorylation within mitochondria to generate ATP.

Among patients with HF, it is intuitive that pump failure may limit bulk transport of oxygenated blood to the periphery. However, it is important to emphasize that abnormalities may exist at multiple points in the oxygen cascade among patients with HF. A detailed analysis of these abnormalities is beyond the scope of this review. However, Table 1 describes critical abnormalities that may exist among patients with HF limiting ${\dot{V}}_{O_{2}}$ and contributing to HF symptoms, impairments in HRqOL and impairments in exercise capacity.

**TABLE 1:** Pathophysiological considerations along the oxygen cascade that contribute to HF symptoms, impairments in HRqOL and impaired exercise capacity among patients with HF.

Pulmonary	Increased pulmonary arterial pressures (Buchanan et al., 2024; Edward, Banchs, et al., 2023, Edward, Parker, et al., 2023; Poole et al., 2012) ${\dot{V}}_{A}$/$\dot{Q}$ mismatching (Poole et al., 2012) Pulmonary congestion (Buchanan et al., 2024; Edward, Banchs, et al., 2023, Edward, Parker, et al., 2023; Sarma et al., 2023) Expiratory flow limitation (Agostoni et al., 2002; Johnson et al., 2000) Ventilatory inefficiency (Agostoni et al., 2002; Johnson et al., 2000) Respiratory steal (Johnson, 2000; Olson et al., 2010)
Cardiovascular	Pump failure (Buchanan et al., 2024; Edward, Banchs, et al., 2023, Edward, Parker, et al., 2023; Sailer et al., 2021) Impaired SV reserve (Buchanan et al., 2024; Edward, Banchs, et al., 2023, Edward, Parker, et al., 2023; Sailer et al., 2021) Sympathetic nerve hyperactivity (Barretto et al., 2009; Mancini et al., 1991; Middlekauff & Sinoway, 2007) Endothelial dysfunction (Drexler et al., 1992; Middlekauff et al., 2004; Poole et al., 2012)
Haematological	Anaemia (Anand & Gupta, 2018)
Musculoskeletal	Impaired vasodilatory reserve (Sarma & Levine, 2018) Sarcopenia (Delp et al., 1997; Esposito et al., 2010; Gielen et al., 2005; Hambrecht et al., 1995; Xu et al., 1998) Decreased type 1 muscle fibres (Behnke et al., 2004) Increased type 2 muscle fibres (Behnke et al., 2004) Enhanced exercise pressor reflex (Barretto et al., 2009; Middlekauff & Sinoway, 2007) Impaired functional sympatholysis (Sarma & Levine, 2018) Decreased capillary density (Cohn et al., 1984; Richardson et al., 1993)
Mitochondrial	Mitochondrial dysfunction (Delp et al., 1997; Esposito et al., 2010; Gielen et al., 2005; Hambrecht et al., 1995; Xu et al., 1998) Decreased enzyme activity (Delp et al., 1997; Esposito et al., 2010; Gielen et al., 2005; Hambrecht et al., 1995; Xu et al., 1998) Decreased mitochondrial volume density (Delp et al., 1997; Esposito et al., 2010; Gielen et al., 2005; Hambrecht et al., 1995; Xu et al., 1998)

*Note*: Supporting references are denoted next to each item. Abbreviations: HF, heart failure; HRqOL, health‐related quality‐of‐life; $\dot{Q}$, flow; SV, stroke volume; ${\dot{V}}_{A}$, alveolar ventilation.

## 3 AUTONOMIC AND NEUROMUSCULAR RESPONSE TO EXERCISE

The autonomic nervous system plays a critical role in regulating ${\dot{Q}}_{C}$ during exercise, ensuring that the supply of blood to exercising organs is closely matched to metabolic demand and that blood pressure (BP) is maintained, not falling due to peripheral vasodilatation within exercising muscle (Grotle et al., 2020). The primary mechanisms by which the autonomic nervous system contribute to ${\dot{Q}}_{C}$ control during exercise include central command, the exercise pressor reflex (EPR) and the arterial baroreceptor reflex (Grotle et al., 2020; Williamson, 2010).

### 3.1 Central command

‘Central command’ refers to a feedforward signal originating from high motor cortical centres in the central nervous system (Sarma et al., 2021; Victor et al., 1995). Originally described well over a century ago (Krogh & Lindhard, 1913), central command operates in conjunction with peripheral feedback pathways (e.g., group IV afferent signals, aka ‘metaboreceptors’) and the arterial baroreflex to regulate the cardiovascular response to exercise (Williamson, 2015). The centre of origin for this network is incompletely described (Williamson, 2010, 2015), but operates prior to and at the onset of exercise, coordinating parasympathetic withdrawal and increases in sympathetic outflow (Sarma et al., 2021; Secher, 2007). As described by the late Jere Mitchell over 30 years ago, multiple factors contribute to the role of central command in controlling cardiovascular function during exercise, including type, intensity and duration of exercise, as well as the efficacy of blood flow in meeting metabolic demand of exercising muscle (Williamson, 2010). Additional factors, including somatosensory signals, which send biochemical and mechanical information to the medulla and motor cortex, respectively, also contribute to the perception of effort, influencing cardiovascular function during exercise (Martin et al., 2008; Williamson, 2010). These multiple factors work together to coordinate acceleration in heart rate (HR), maintenance of BP during exercise, as well as increases in cardiac output and delivery of oxygenated blood to exercising muscle (Williamson, 2010).

### 3.2 The EPR

Accumulating evidence suggests that skeletal muscle abnormalities play a major role in exercise limitations associated with HF (Grotle et al., 2020). Alterations in function of skeletal muscle afferent fibres lead to impairments in exercise ${\dot{Q}}_{C}$ and normal regulation of BP during exercise. These afferent fibres are described by the EPR, a feedback mechanism arising from exercising muscle, modulating sympathetic tone to further adjust ${\dot{Q}}_{C}$ to match supply with metabolic demand of exercising muscle (Grotle et al., 2020; Williamson, 2010). The two primary components of the EPR are group III and group IV skeletal muscle afferent fibres (Grotle et al., 2020). Group III afferents are thinly myelinated fibres responding to mechanical stimuli and are activated at the onset of exercise in proportion to the degree of motion or tension of exercising skeletal muscle. These mechanoreceptors activate very quickly, within 200 ms of the onset of exercise, and remain active throughout the duration of effort. Group IV metaboreceptors are unmyelinated afferent fibres, responding to metabolic stimuli. These fibres become active over time, up to 30 s after the initiation of exercise as a result of buildup of metabolic byproducts of exercise. Both group III and IV fibres, acting together or in isolation, increase ${\dot{Q}}_{C}$ and BP by increasing sympathetic nerve activity (Kaufman et al., 1983; Mitchell et al., 1983). The mechanoreflex is incompletely understood, since limitations in technology preclude identification of exact receptors and channels (Grotle et al., 2020). Metaboreceptors are activated by metabolic or chemical stimuli that accumulate locally as a byproduct of exercise, and include such factors as lactic acid, ATP, bradykinin, hydrogen ions and capsaicin (Grotle et al., 2020).

Muscle mechanoreceptor activity is elevated among patients with HF with reduced ejection fraction (HFrEF) (Middlekauff et al., 2004; Middlekauff & Sinoway, 2007). In a very elegant study using microneurography to record muscle sympathetic nerve activity (MSNA), patients with HFrEF (*n* = 12, left ventricular ejection fraction (LVEF) 26 ± 2%) and healthy controls (*n* = 13) underwent rhythm handgrip exercise at 20% maximal voluntary contraction to stimulate mechanoreceptors (Middlekauff et al., 2004). Compared to the healthy controls, individuals with HFrEF demonstrated an early rise in MSNA during handgrip exercise, consistent with an enhanced mechanoreflex resulting from a heightened basal sensitivity to mechanical stimuli (Middlekauff et al., 2004). It has been suggested that this increase in mechanoreceptor sensitivity may be compensatory, since increased sympathetic nerve activity may stimulate a failing LV to increase ${\dot{Q}}_{C}$ (Grotle et al., 2020). However, over time, a downward cycle may ensue, whereby low ${\dot{Q}}_{C}$ and impaired skeletal muscle blood flow further activate mechanoreceptors, contributing to early fatigue and impaired exercise capacity (Grotle et al., 2020).

Data are conflicting as to whether the metaboreflex is accentuated or blunted among patients with HFrEF (Grotle et al., 2020). In an analysis involving both passive and dynamic lower extremity exercise (to activate metaboreceptors) of patients with HFrEF, dynamic exercise followed by post‐exercise circulatory arrest led to increased ventilation, suggestive of metabolic stimulation of the muscle metaboreceptors (Scott et al., 2000). In another analysis of metaboreflex function among patients with HFrEF and healthy controls, both groups demonstrated a similar increase in MSNA and exercise pressor responses to static handgrip (Sterns et al., 1991). However, MSNA quickly normalized during post‐exercise circulatory arrest among the HFrEF group, whereas MSNA remained elevated among controls, suggesting impaired metaboreflex function among individuals with HFrEF (Sterns et al., 1991). This impairment in metaboreceptor function limits augmentations in ${\dot{Q}}_{C}$ and BP that otherwise occur during exercise due to a buildup of metabolic byproducts, contributing to limitations in exercise tolerance.

## 4 CARDIOVASCULAR PERFORMANCE DURING EXERCISE

### 4.1 Cardiac output and its determinants: HR and stroke volume

HR increases during exercise due to the aforementioned increase in sympathetic activity and parasympathetic withdrawal (Rudofker et al., 2025). In healthy individuals, HR increases with ${\dot{V}}_{O_{2}}$ uptake. As exercise intensity increases, the rate of increase in HR begins to level off, particularly as an individual's maximum exercise capacity is approached. This deflection point in HR is referred to as the Conconi HR and coincides with the onset of blood lactate accumulation (Conconi et al., 1996). The rate of increase in HR may vary between individuals according to level of fitness and the presence/absence of a cardiomyopathy. For example, sedentary/deconditioned individuals typically experience rapid increases in HR with the onset of exercise, whereas HR increase is more gradual among fit individuals (Gaffney et al., 1985).

‘Maximum predicted heart rate’ (MPHR) refers to the highest HR that may be achieved during a symptom‐limited exercise test. MPHR may be calculated by a number of different equations, but is most often determined by the equation: MPHR = 220 − age (years). This equation is undoubtedly imprecise and inherently limited by its inability to account for the variability that exists between individuals. However, it provides a general target that an individual's HR should theoretically be able to achieve. One traditional criterion for determining maximal effort on a stress test is arbitrary threshold of achieving 85% MPHR (Rudofker et al., 2025). Chronotropic incompetence (CI) refers to the inability of the HR to increase appropriately in response to physical activity or metabolic demand (Zweerink et al., 2018). More specifically, CI has been defined as an inability to achieve 80% MPHR (Zweerink et al., 2018).

However, there are a number of reasons an individual's HR may not increase as expected during exercise, such as effort (or lack thereof), inappropriate protocol selection during formal exercise testing, medications or true pathology. In an analysis of HFpEF patients with CI who were also treated with beta‐blockers, removal of the beta‐blocker led to an improvement in ${\dot{V}}_{O_{2}\max}$ by ∼2 mL/kg/min and an improvement in peak HR during exercise (from 97 to 127 bpm) (Palau et al., 2021). However, for patients with HFpEF and CI, implantation of a pacemaker does not improve functional capacity or HRqOL. In the RAPID‐HF trial, HFpEF patients with CI underwent pacemaker implantation followed by randomization to atrial rate responsive pacing vs. no pacing in a double‐blind, randomized crossover protocol (Reddy et al., 2023). Pacing led to an increase in peak exercise HR but this was offset by a reduction in exercise stroke volume (SV) and as a result, there was no improvement in exercise ${\dot{Q}}_{C}$ or ${\dot{V}}_{O_{2}\max}$ (Reddy et al., 2023).

Generally, maximal SV during exercise is achieved at a relatively low, submaximal level of exercise (Cornwell et al., 2020), approximately 50% of an individual's ${\dot{V}}_{O_{2}\max}$ (Sarma & Levine, 2018). This inability to further augment SV at higher levels of exercise is related to pericardial constraint, limiting further increases in end‐diastolic volume. Elegant studies from animal models and humans with HFpEF suggest that increases in SV and ${\dot{Q}}_{C}$ can be achieved by removal of the pericardium, for example, in healthy dogs (*n* = 10) completed exercise testing prior to and following pericardiectomy (Stray‐Gundersen et al., 1986). Following pericardiectomy, there was no change in peak exercise HR; however, peak ${\dot{Q}}_{C}$, SV and ${\dot{V}}_{O_{2}\max}$ were all increased compared to baseline conditions (Stray‐Gundersen et al., 1986). In an elegant study of humans with HFpEF, pericardiotomy performed as part of a surgically indicated procedure (e.g., aortic valve replacement, coronary artery bypass grafting) led to a reduction in pulmonary capillary wedge pressure (PCWP) during volume loading of the LV with either saline infusion or a leg raise (Borlaug et al., 2018). These types of studies suggest that pericardial constraint limits further increases in exercise SV that would otherwise occur, and contributes to increases in filling pressures among patients with HFpEF.

### 4.2 Left ventricular haemodynamics and pulmonary arterial pressures

Resting and exertional haemodynamics among healthy individuals are well characterized (Kovacs et al., 2009). In a systematic review of healthy individuals (*n* = 1187) from 47 studies, supine resting mean PAP (mPAP) was 14.0 ± 3.3 mmHg and PCWP was 8.0 ± 2.9 mmHg (Kovacs et al., 2009). During exercise, mPAP increased in response to intensity of exercise with peak value of 25.6 ± 5.6 mmHg and was also higher among older individuals above 50 years of age (Kovacs et al., 2009). Exercise PCWP increased to 14.9 ± 7.9 mmHg and was also higher among older individuals above 50 years of age (Kovacs et al., 2009). ${\dot{Q}}_{C}$ generally increases by three to five‐fold from rest to peak effort (Cornwell et al., 2020). Haemodynamic values obtained during exercise are best understood when placed in context of exercise ${\dot{Q}}_{C}$. A mPAP/${\dot{Q}}_{C}$ slope >3 mmHg/L/min is used as a criterion for diagnosis of exercise pulmonary hypertension, and importantly, is associated with reduced exercise capacity and overall survival (Lewis et al., 2011; Naeije et al., 2013). A PCWP/${\dot{Q}}_{C}$ slope >2 mmHg/L/min is considered abnormal, commonly seen among patients with HFpEF (Eisman et al., 2018) and HFrEF (Edward, Banchs, et al., 2023, Edward, Parker, et al., 2023), and is also associated with a reduced exercise capacity and survival (Eisman et al., 2018).

**FIGURE 2:**
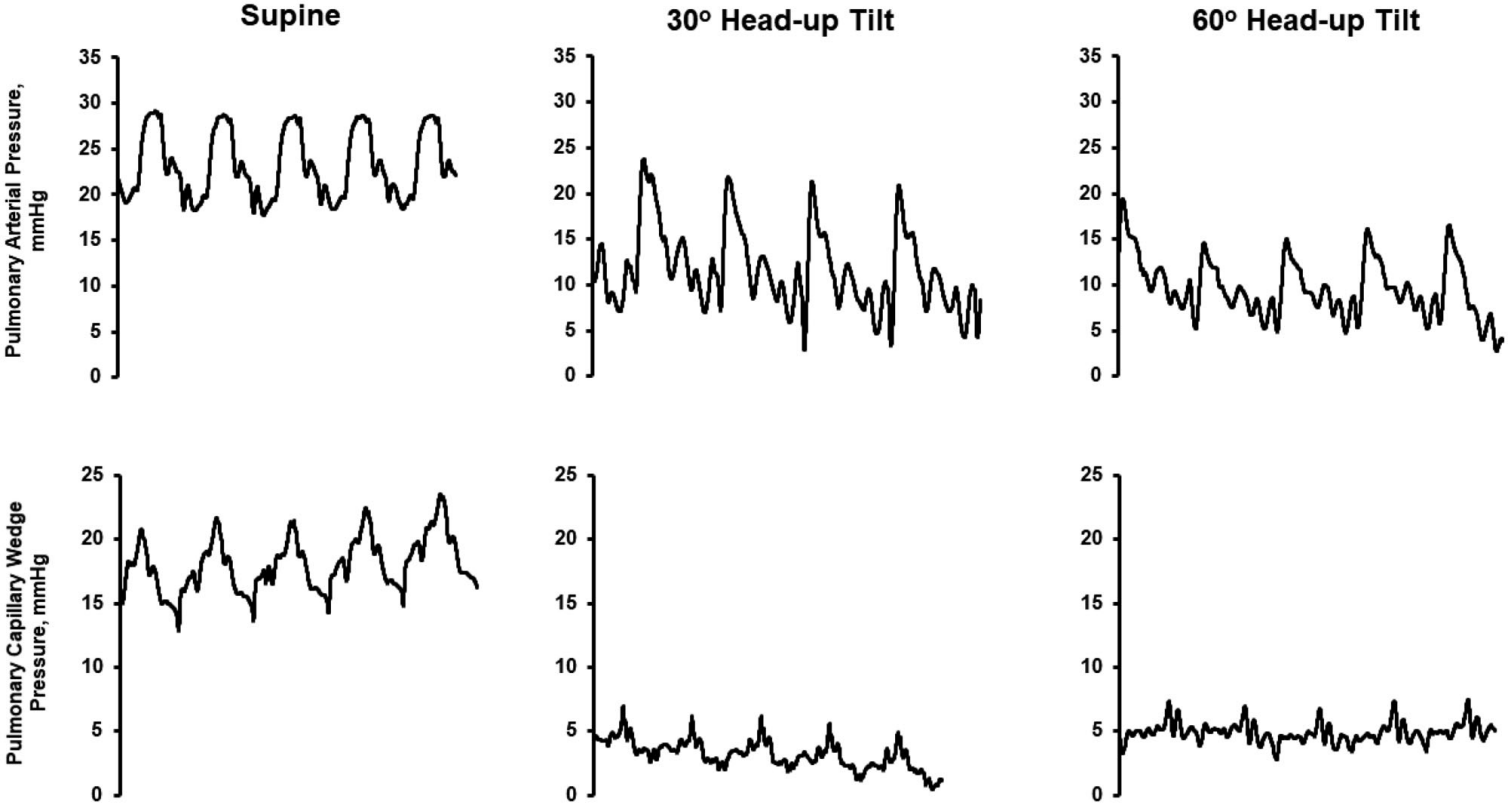
Example tracings of invasive haemodynamics during a progressive head‐up tilt in a 73‐year‐old female with HFrEF (LVEF 30%). Data obtained from Swan–Ganz catheter demonstrating pulmonary arterial pressure and pulmonary capillary wedge pressure at supine, 30° and 60° head‐up tilt. The increased gravitational gradient associated with tilt leads to reduction in filling pressures. Data provided from senior author's laboratory. HF, heart failure; HFrEF, HF with reduced ejection fraction; LVEF, left ventricular ejection fraction.

It is also important to account for body position when determining resting and exertional haemodynamics and the overall response to exercise. Upright posturing introduces a gravitational load on the cardiovascular system, leading to a downward displacement of fluid and reducing ventricular filling pressures and PAP (Figure 2) (Berlier et al., 2022; Buchanan et al., 2024; Kovacs et al., 2009). These reductions in filling pressures, with subsequent drop in upright SV and ${\dot{Q}}_{C}$, also contribute to orthostatic intolerance among these patients (Reddy, 2024; Soloveva et al., 2024). The skeletal muscle pump is more effective at promoting venous return in the upright position during exercise due to greater recruitment of skeletal muscle than during supine exercise (Leyk et al., 1994). However, in the supine position, a less effective muscle pump is offset by higher filling pressures. In addition, the determinants of exercise ${\dot{Q}}_{C}$ vary depending on body posture (Forbes et al., 2023; Leyk et al., 1994). Generally, ${\dot{Q}}_{C}$ is higher during supine rest than upright rest (Berlier et al., 2022; Leyk et al., 1994). In the upright position, there is a compensatory increase in resting HR (Berlier et al., 2022). Peak exercise ${\dot{Q}}_{C}$ is generally similar between supine and upright exercise, but the mechanisms by which ${\dot{Q}}_{C}$ is achieved vary by position, since SV is greater during supine exercise, while peak HR is greater during upright exercise (Berlier et al., 2022). During supine exercise, lactate concentration generally increases faster, and maximal power and exercise duration are lower compared to upright exercise (Berlier et al., 2022; Leyk et al., 1994). Haemodynamic assessments during exercise are crucial to understanding mechanisms of exertional dyspnoea and appropriately diagnosing HFpEF. Among patients undergoing supine invasive exercise testing, an increase in PCWP to 25 mmHg or more is consistent with HFpEF. Among patients with exertional dyspnoea and LVEF > 50% undergoing invasive haemodynamic assessment during supine exercise, individuals with HFpEF experienced rapid increases in filling pressures, with a peak PA and PCWP of 43 ± 7 and 32 ± 6 mmHg, respectively (Borlaug et al., 2010). In that same study, individuals evaluated with non‐cardiac causes of dyspnoea had a peak PA and PCWP of 23 ± 5 and 13 ± 5 mmHg, respectively, demonstrating the severity of exertional haemodynamics among those with HFpEF (Borlaug et al., 2010). However, it is not clear that haemodynamic abnormalities alone are the sole, or primary cause of impairments in exercise capacity among patients with HFpEF. In another haemodynamic assessment, HFpEF patients (*n* = 30) completed invasive haemodynamic assessment during upright seated exercise while receiving sublingual nitroglycerin or placebo in a randomized, single‐blind, crossover pattern (Sarma et al., 2023). During exercise with placebo, PCWP increased from 8 ± 4 at rest to 35 ± 9 mmHg at peak exercise (Sarma et al., 2023). Exercise with nitroglycerin reduced submaximal PCWP and peak PCWP by 5 ± 5 and 7 ± 6 mmHg, respectively. However, the ${\dot{V}}_{O_{2}\mathrm{peak}}$ with nitroglycerin was unchanged when compared to placebo (11.8 ± 3.7 vs. 11.9 ± 3.9 mL/kg/min) and there was no difference in peak exertional SV or ${\dot{Q}}_{C}$ (Sarma et al., 2023). In this study, it should be noted that while nitroglycerin administration reduced peak exercise PCWP, the effect was modest, as the peak PCWP was still markedly abnormal at 29 ± 9 mmHg. It remains to be seen if greater reductions in exercise PCWP would lead to improvements in exercise capacity among these patients. However, the rate of perceived breathlessness during exercise was surprisingly worse with nitroglycerin compared to placebo (4.03 ± 2.18 vs. 3.43 ± 1.94, *P* = 0.009) and the reduction in PCWP also increased ventilation–perfusion (${\dot{V}}_{A}$/$\dot{Q}$) mismatching, increased the ${\dot{V}}_{E}$/${\dot{V}}_{CO_{2}}$ slope (ratio of minute ventilation (${\dot{V}}_{E}$) and carbon dioxide production (${\dot{V}}_{CO_{2}}$)) and worsened the alveolar–arterial $P_{O_{2}}$ difference ($A-aD_{O_{2}}$ gradient) (Balmain et al., 2023).

These types of observations suggest that among patients with HFpEF, an elevated PCWP may not appear to be the primary mechanism limiting exercise capacity (Borlaug et al., 2018). In the INDIE‐HFpEF study, patients with HFpEF (*n* = 105) were randomized to receive inorganic nitrites in a double‐blind, placebo‐controlled crossover fashion (Borlaug et al., 2018). ${\dot{V}}_{O_{2}\max}$ (mean [standard deviation] for nitrite vs. placebo: 13.5 [3.3] vs. 13.7 [3.5] mL/kg/min) did not improve with inorganic nitrites (Borlaug et al., 2018). In REDUCE LAP‐HF I, patients with HFpEF (*n* = 44) were randomized to undergo interatrial shunt device versus sham procedure (Feldman et al., 2018). While shunt placement reduced PCWP compared to sham (peak PCWP decreased by 3.5 ± 6.4 mmHg), peak exercise workload was not improved (Feldman et al., 2018).

Emerging data indicate that HFpEF patients suffer from both central and peripheral limitations when trying to exercise. In an analysis of HFpEF (*n* = 45) patients undergoing invasive haemodynamic exercise testing, when individuals were stratified according to the ${\dot{Q}}_{C}$/${\dot{V}}_{O_{2}}$ relationship during exercise testing, approximately two‐thirds of patients were peripherally limited, as evidenced by a ${\dot{Q}}_{C}$/${\dot{V}}_{O_{2}}$ slope greater than 5–6, while only one‐third of patients were centrally limited, as evidenced by a ${\dot{Q}}_{C}$/${\dot{V}}_{O_{2}}$ slope less than 5–6 (Skow et al., 2024). Patients with a peripheral limitation had a lower $A{\dot{V}}_{O_{2}\mathrm{diff}}$ than those with central limitations (11.1 ± 1.6 vs. 13.5 ± 2.0 mL O_2_/dL), as well as lower skeletal muscle oxygen utilization (Skow et al., 2024). Thus, while patients with HFpEF are limited by markedly abnormal LV and PA haemodynamics, peripheral limitations including impaired oxygen utilization also contribute to hallmark symptoms and reduced exercise capacity.

**FIGURE 3:**
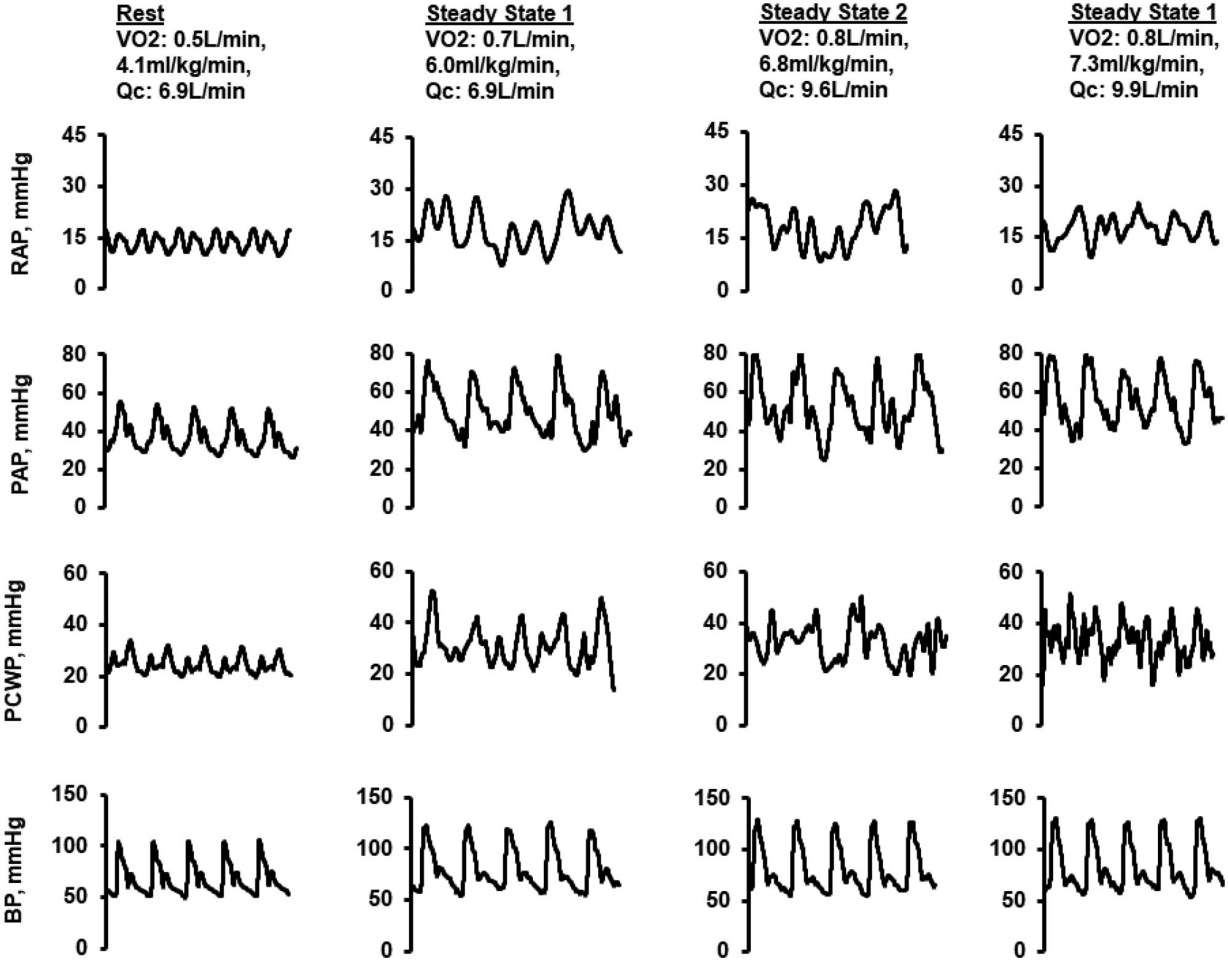
Haemodynamic tracings obtained from a 67‐year‐old man with ischaemic cardiomyopathy (LVEF 23%), undergoing invasive cardiopulmonary exercise testing. Data acquired during rest, two levels of submaximal exercise, as well as peak effort. Data provided by senior author (Edward, Banchs, et al., 2023, Edward, Parker, et al., 2023). BP, blood pressure; LVEF, left ventricular ejection fraction; PAP, pulmonary arterial pressure; PCWP, pulmonary capillary wedge pressure.

Patients with HFrEF also suffer from marked increases in left‐sided and PA pressures during exercise (Figure 3) (Edward, Banchs, et al., 2023, Edward, Parker, et al., 2023). Among HFrEF patients (*n* = 35, LVEF 23 ± 8%) undergoing invasive cardiopulmonary exercise testing, patients experienced large increases in mPAP and PCWP during the transition from rest to submaximal exercise, with further increases at peak effort (Edward, Banchs, et al., 2023, Edward, Parker, et al., 2023). ${\dot{Q}}_{C}$ and SV were also severely blunted in these patients, with a less than two‐fold increase in ${\dot{Q}}_{C}$ from rest to peak effort.

### 4.3 Right ventricular function

Knowledge of right ventricular (RV) function has historically lagged behind that of the LV (Brener et al., 2021; Cornwell III & Buttrick, 2023; Cornwell et al., 2020; Edward, Banchs, et al., 2023, Edward, Parker, et al., 2023; Houston et al., 2023; Konstam et al., 2018). The RV has even been referred to as a ‘forgotten ventricle’ (Amsallem et al., 2018) or a passive conduit (Dell'Italia, 2012). However, several elegant non‐invasive and invasive haemodynamic assessments in the last decade have provided a wealth of information regarding RV performance in multiple populations, including normal healthy individuals (Cornwell et al., 2020), endurance athletes (Edward et al., 2022; La Gerche et al., 2012; Oxborough et al., 2011), HFpEF (Rommel et al., 2018), HFrEF (Edward, Banchs, et al., 2023, Edward, Parker, et al., 2023) and HFrEF patients supported by mechanical circulatory support devices (aka, left ventricular assist devices) (Tran et al., 2021), and patients with pulmonary vascular disease (Hsu et al., 2016; Richter et al., 2021; Tedford et al., 2013). These studies demonstrate the centrality and role of the RV during exercise, mechanisms by which RV performance is impaired in diseased states, as well as the impact of overt impairments in RV contractile and lusitropic reserve on overall exercise capacity.

**FIGURE 4:**
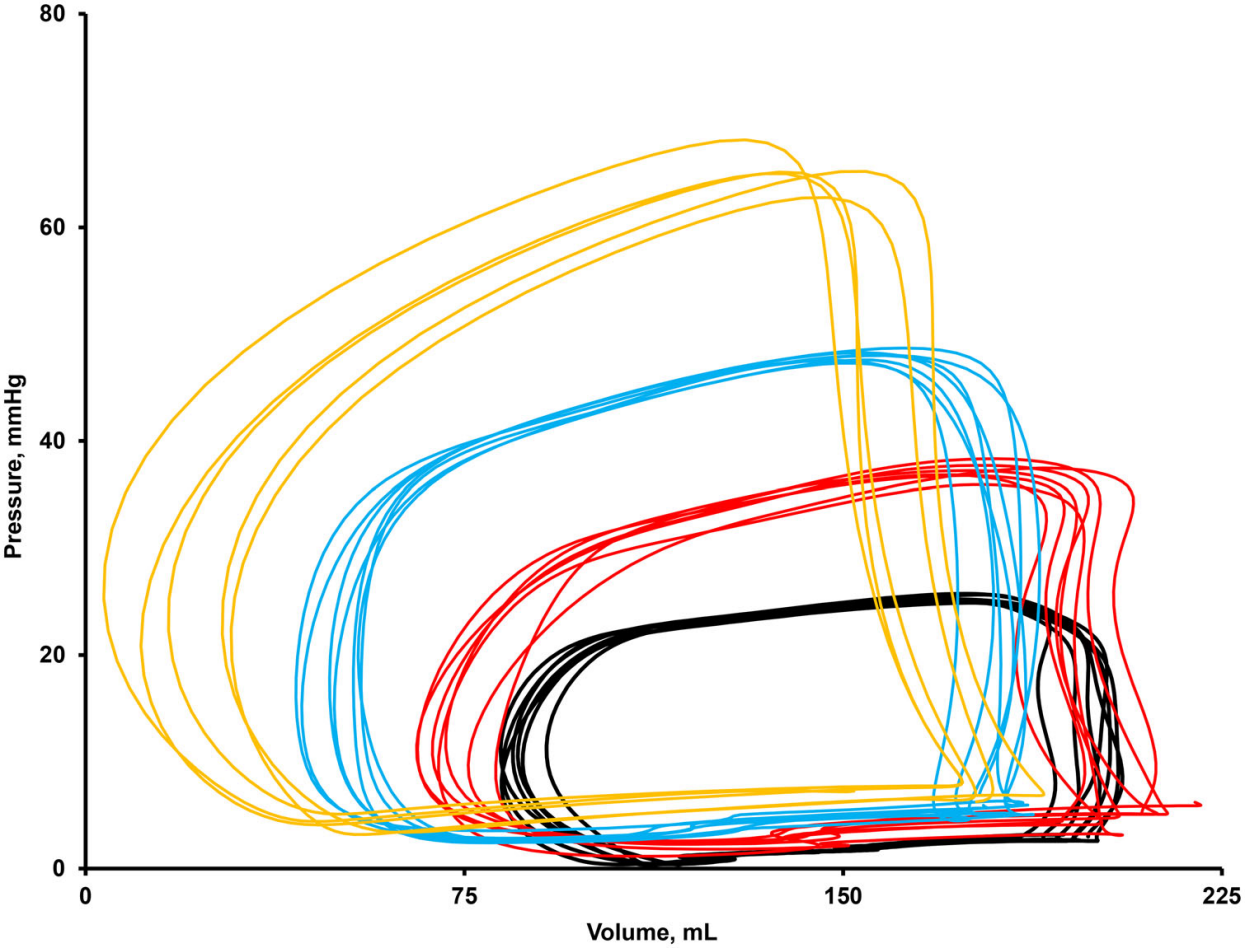
Example tracings of real‐time right ventricular pressure–volume loops during exercise from a healthy individual completing invasive cardiopulmonary exercise testing with a conductance catheter during upright cycle ergometry. Black represents resting loops, red and blue represent two levels of submaximal exercise, and gold represents peak exercise. Data provided by senior author (Cornwell et al., 2020).

In the normal healthy heart, the RV has an enormous amount of contractile and lusitropic reserve (Figure 4) (Cornwell et al., 2020; Edward, Banchs, et al., 2023, Edward, Parker, et al., 2023). As such, the RV works in conjunction with the skeletal muscle pump and local vasodilatory forces to promote venous return and ventricular filling during diastole and increases contractile forces to propel blood into the pulmonary arterial vasculature despite increases in afterload (i.e., higher PAP). Thus, the RV actively contributes to overall cardiac performance during all phases of the cardiac cycle during exercise.

In contrast, among patients with HF, the RV may suffer from impairments in contractile reserve and/or increased stiffness. In an analysis of RV diastolic function during invasive handgrip exercise, patients with HFpEF demonstrated an upward shift in the RV end‐diastolic pressure–volume curve, indicative of increased RV stiffness (Rommel et al., 2018). In this same study, the RV end‐diastolic pressure–volume curve was not increased among controls (Rommel et al., 2018). While it is well‐known that HFpEF patients suffer from increased stiffness of the LV, the upward shift in the RV end‐diastolic pressure–volume curve with exercise indicates that HFpEF is a biventricular disease, and impairments in cardiac performance are not due solely due a stiff LV (Edward, Banchs, et al., 2023, Edward, Parker, et al., 2023).

Among patients with HFrEF, the RV is limited by impairments in contractile reserve, with minimal augmentation in SV and ${\dot{Q}}_{C}$ during submaximal and peak exercise (Edward, Banchs, et al., 2023, Edward, Parker, et al., 2023). These abnormalities, in combination with an abnormal rise in ventricular and PA filling pressures during exercise, lead to additional haemodynamic aberrations brought on during exercise adversely impacting pulmonary arterial compliance (PAC) and pulmonary vascular resistance (PVR). PAC is defined as:

$$\mathrm{PAC}=\mathrm{SV}/\left(\mathrm{PAS}-\mathrm{PAD}\right)$$

where SV is stroke volume, PAS is systolic pulmonary arterial pressure and PAD is diastolic pulmonary arterial pressure.

PVR is defined as:

$$\mathrm{PVR}=\left(\mathrm{mPAP}-\mathrm{PCWP}\right)/{\dot{Q}}_{C}$$

where mPAP is mean pulmonary arterial pressure, PCWP is pulmonary capillary wedge pressure and ${\dot{Q}}_{C}$ is cardiac output.

Clinically, PAC and PVR can both be used to describe RV afterload and are inversely related to each other, such that a higher RV afterload is typified by an increased PVR and reduced PAC (Lankhaar et al., 2008; Saouti et al., 2010). In an analysis of 35 patients with HFrEF (51% non‐ischaemic, LVEF 23 ± 8%, tricuspid annular plane systolic excursion 1.8 ± 0.5 cm) undergoing invasive cardiopulmonary exercise testing with pulmonary arterial catheters, PAC declined during exercise due to a blunted augmentation of SV and a large increase in PAS (Edward, Banchs, et al., 2023, Edward, Parker, et al., 2023). The PVR response may be variable depending on the change in transpulmonary gradient during exercise (i.e., mPAP − PCWP) in relation to the degree of increase in ${\dot{Q}}_{C}$. Among patients with mild HFrEF, modest increases in transpulmonary gradient are offset by larger increase in ${\dot{Q}}_{C}$, causing PVR to drop; however, among patients with more severe HFrEF, large increases in the transpulmonary gradient outweigh very small increases in ${\dot{Q}}_{C}$, causing PVR to rise (Edward, Banchs, et al., 2023, Edward, Parker, et al., 2023). These invasive haemodynamic observations reflect the larger trend in right‐sided haemodynamics among patients with HFrEF, namely, that a blunted contractile reserve combined with an increased RV afterload impairs the ability to maintain ventricular–arterial (VA) coupling, particularly among patients with severe HFrEF (Edward, Banchs, et al., 2023, Edward, Parker, et al., 2023).

Patients with a Fontan circulation serve as a case‐in‐point for the role of the RV during exercise. The Fontan procedure is a palliative (not curative) operation for individuals born with a single ventricle, a type of cyanotic congenital heart disease. This procedure involves creation of an artificial cavopulmonary connection, directing blood returning from the periphery through the vena cava into the pulmonary arterial circulation and preventing mixing of oxygen‐poor ‘blue blood’ with oxygen‐rich ‘red blood’. In the normal circulation, blood returning to the heart is propelled into the pulmonary arterial circulation by a contracting RV. However, in a Fontan circuit, the LV is responsible for propelling blood into the systemic circulation *and in the absence of a contracting RV*, must also propel deoxygenated blood into the pulmonary vascular bed. While the systemic muscle pump may provide modest contributions of flow into the lungs, the LV is required for bulk transit of systemic and pulmonary flow (Mahendran et al., 2023). As a result, patients with a Fontan circuit suffer from several abnormalities during exercise, including a ${\dot{V}}_{O_{2}\max}$ ∼55–65% predicted (Atz et al., 2017; Egbe et al., 2017; Fernandes et al., 2011; Mahendran et al., 2023), CI (Gewillig et al., 1990), systemic hypoxaemia due to persistent Fontan fenestration or shunting, reduced breathing reserve, impaired ventilatory efficiency and exercise oscillatory ventilation (Mahendran et al., 2023; Nathan et al., 2015). Furthermore, less than one‐third of these patients have a ${\dot{V}}_{O_{2}\max}$ that is in the range of normal or supranormal (Mahendran et al., 2023; Powell et al., 2020; Weinreb et al., 2020). Thus, the absence of a normally functioning RV leads to cardiac and pulmonary vascular abnormalities that limit overall exercise capacity.

## 5 CONCLUSION

Multiple factors contribute to ${\dot{Q}}_{C}$ during exercise. Central command, in combination with EPR and the arterial baroreceptors, works to tightly match ${\dot{Q}}_{C}$ to the metabolic demand of exercising muscle and also maintain arterial perfusion pressure. LV and RV contractility increase to maintain VA coupling and maintain forward flow through the systemic and pulmonary vascular beds. At least among healthy populations, ${\dot{Q}}_{C}$ increases by ∼6 L/min for every 1 L/min increase in ${\dot{V}}_{O_{2}}$. Among patients with HF, however, these reflexes and relationships may break down, contributing to fatigue, impairments in HRqOL, dyspnoea on exertion and reduced exercise capacity. More specifically, abnormalities exist in the EPR including accentuation of group III afferents. Group IV afferent activity is variably impacted by HF. Cardiovascular haemodynamics are markedly abnormal, with large increases in filling pressures and limited ventricular contractile reserve. While advancements in ‘guideline‐directed medical therapy’ have led to improvements in survival among these patients, future studies are needed to explore novel methods of optimizing exertional cardiovascular performance to improve functional capacity and minimize symptom burden.

## AUTHOR CONTRIBUTIONS

All authors contributed to the conception of this review article and contributed to drafting the work or critical revision for intellectual content. The authors have approved the final version of the manuscript and agree to be accountable for all aspects of the work in ensuring that questions related to the accuracy or integrity of any part of the work are appropriately investigated and resolved. All persons designated as authors qualify for authorship and those who qualify for authorship are listed.

## CONFLICT OF INTEREST

None declared.

## FUNDING INFORMATION

No funding was received for this work.
